# PRESIDENTIAL SYMPOSIUM: BUILDING BRIDGES ACROSS THE SCIENCES TO CATALYZE SLEEP DISPARITIES RESEARCH

**DOI:** 10.1093/geroni/igad104.0664

**Published:** 2023-12-21

**Authors:** Karen Lincoln, Karen Lincoln

## Abstract

This BSS Presidential Symposium brings together scholars from Sociology, Social and Family Dynamics, Human Development and Family Studies, Occupational Science and Medicine to highlight a multidisciplinary approach to sleep disparities research. Sleep is essential for restoration of healthy brain function, the immune system, and overall physical and emotional well-being. Conversely, sleep deficiencies are associated with a wide range of poor physical and emotional health outcomes, health behaviors and premature mortality. Strong empirical evidence indicates that sleep health is socially patterned, such that minoritized populations are generally more likely to experience sleep disruptions, disorders, and difficulties. These sleep deficiencies are also observed across indicators of socioeconomic status, such as income and educational attainment. Despite documentation of sleep disparities, studies that examine underlying health disparity causal pathways contributing to sleep health disparities are limited. This symposium is aligned with recommended strategies to address sleep health disparities from the National Institutes of Health, including: a) develop and promote integrative research on sleep health disparities, b) investigate the health and social causes and consequences of sleep health disparities, and c) develop multilevel, culturally tailored interventions to address sleep health disparities. By placing people and place at the center of their research, these scholars demonstrate their approach to addressing current research gaps, challenges and opportunities in sleep and health disparities research among middle-aged and older adults that are important to advance this emerging field.

